# Supplementary material for: Machine Learning Accelerates Crystallization for Structure Determination
Source: Angew Chem Int Ed Engl. 2026 May 4;65(25):e1218503. doi: 10.1002/anie.1218503 (PMC13266958; doi:10.1002/anie.1218503)

checkCIF/PLATON report

Structure factors have been supplied for datablock(s) lcz014

THIS REPORT IS FOR GUIDANCE ONLY. IF USED AS PART OF A REVIEW PROCEDURE FOR PUBLICATION, IT SHOULD NOT REPLACE THE EXPERTISE OF AN EXPERIENCED CRYSTALLOGRAPHIC REFEREE.

No syntax errors found. CIF dictionary Interpreting this report

Datablock: lcz014

Bond precision:	C-C = 0.0096 A		Wavelength=1.54184
Cell:	a=8.4877(1)	b=40.1219(4)	c=11.0437(1)
	alpha=90	beta=96.543(1)	gamma=90
Temperature:	100 K		

	Calculated	Reported
Volume	3736.36(7)	3736.35(7)
Space group	P 21/c	P 1 21/c 1
Hall group	-P 2ybc	-P 2ybc
Moiety formula	C15 H3 Ag3 F18 N6, 2(C8 H8 O2)	C15 H3 Ag3 F18 N6, 2(C8 H8 O2)
Sum formula	C31 H19 Ag3 F18 N6 O4	C31 H19 Ag3 F18 N6 O4
Mr	1205.13	1205.13
Dx,g cm-3	2.142	2.142
Z	4	4
Mu (mm-1)	13.769	13.769
F000	2328.0	2328.0
F000’	2338.98	
h,k,lmax	10,50,14	10,51,13
Nref	8030	7710
Tmin,Tmax	0.240,0.252	0.253,1.000
Tmin’	0.051	

Correction method= # Reported T Limits: Tmin=0.253 Tmax=1.000
AbsCorr = MULTI-SCAN

Data completeness= 0.960   Theta(max)= 78.401

R(reflections)= 0.0514(6870)   wR2(reflections)= 0.1403(7710)

S = 1.070   Npar= 591

The following ALERTS were generated. Each ALERT has the format
test-name_ALERT_alert-type_alert-level.
Click on the hyperlinks for more details of the test.

Alert level C

```
PLAT213_ALERT_2_C Atom F2              has ADP max/min Ratio .....        3.4 prolat
PLAT213_ALERT_2_C Atom F3A             has ADP max/min Ratio .....        3.1 prolat
PLAT220_ALERT_2_C NonSolvent   Resd 1  F   Ueq(max)/Ueq(min) Range        3.3 Ratio
PLAT234_ALERT_4_C Large Hirshfeld Difference F1A      --C01Q     .       0.16 Ang.
PLAT242_ALERT_2_C Low    'MainMol' Ueq as Compared to Neighbors of       C014 Check
PLAT250_ALERT_2_C Large U3/U1 Ratio for <U(i,j)> Tensor(Resd    1)        2.1 Note
PLAT342_ALERT_3_C Low Bond Precision on  C-C Bonds ..............    0.00961 Ang.
PLAT906_ALERT_3_C Large K Value in the Analysis of Variance ......      4.889 Check
PLAT911_ALERT_3_C Missing FCF Refl Between Thmin & STh/L=    0.600         17 Report
                1  0  0,  10  0  0,   0  4  0,  10  1  1,  -1  2  1,   0  2  1,
                9  2  1,  -2  1  2,   9 11  3,  -7 23  3,   9  0  4,   9  1  4,
                9  2  4,  -4  4  4,  -7 21  4,  -8 24  6,  -1  0  8,
PLAT934_ALERT_3_C Number of (Iobs-Icalc)/Sigma(W) > 10 Outliers ..          1 Check
                8  0  2,
```

Alert level G

```
PLAT002_ALERT_2_G Number of Distance or Angle Restraints on AtSite          7 Note
PLAT003_ALERT_2_G Number of Uiso or U(i,j)  Restrained non-H-Atoms          8 Report
PLAT007_ALERT_5_G Number of Unrefined Donor-H Atoms .............           2 Report
              H00L  H00I
PLAT083_ALERT_2_G SHELXL Second Parameter in WGHT  Unusually Large      43.42 Why ?
PLAT142_ALERT_4_G s.u. on b - Axis Small or Missing .............     0.00040 Ang.
PLAT143_ALERT_4_G s.u. on c - Axis Small or Missing .............     0.00010 Ang.
PLAT176_ALERT_4_G The CIF-Embedded .res File Contains SADI Records          3 Report
PLAT177_ALERT_4_G The CIF-Embedded .res File Contains DELU Records          1 Report
PLAT178_ALERT_4_G The CIF-Embedded .res File Contains SIMU Records          1 Report
PLAT186_ALERT_4_G The CIF-Embedded .res File Contains ISOR Records          3 Report
PLAT188_ALERT_3_G A Non-default SIMU Restraint Value has been used     0.0100 Report
PLAT192_ALERT_3_G A Non-default DELU Restraint Value for SecondPar     0.0200 Report
PLAT242_ALERT_2_G Low    'MainMol' Ueq as Compared to Neighbors of       C016 Check
PLAT242_ALERT_2_G Low    'MainMol' Ueq as Compared to Neighbors of       C01D Check
PLAT242_ALERT_2_G Low    'MainMol' Ueq as Compared to Neighbors of       C01K Check
PLAT242_ALERT_2_G Low    'MainMol' Ueq as Compared to Neighbors of       C01Q Check
PLAT301_ALERT_3_G Main Residue  Disorder ..............(Resd    1)         7% Note
PLAT434_ALERT_2_G Short Inter HL..HL Contact F005     ..F007     .       2.84 Ang.
                                                  1+x,y,1+z  =      1_656 Check
PLAT434_ALERT_2_G Short Inter HL..HL Contact F00F     ..F00F     .       2.84 Ang.
                                                1-x,1-y,1-z  =      3_666 Check
PLAT720_ALERT_4_G Number of Unusual/Non-Standard Labels ..........         78 Note
              Ag01    Ag02    Ag03    F004    F005    F006    F007    F008
              F009    F00A    F00B    F00C    F00D    F00E    F00F    O00G
```

```
              F00H    O00I    H00I    O00J    N00K    O00L    H00L    F00M
              N00N    F00O    N00P    N00Q    N00R    N00S    C00U    C00V
              C00W    H00W    C00X    C00Y    C00Z    H00Z    C010    C011
              C012    C013    H013    C014    C015    C016    C017    C018
              C019    C01A    H01A    C01B    H01B    C01C    C01D    C01E
              C01F    H01F    C01G    H01G    C01H    H01H    C01I    H01C
              H01D    H01E    C01J    H01J    C01K    C01M    H01I    H01K
              H01L    C01N    H01N    C01O    H01O    C01Q
PLAT860_ALERT_3_G Number of Least-Squares Restraints ............         63 Note
PLAT883_ALERT_1_G Absent Datum for _atom_sites_solution_primary ..     Please Do !
PLAT910_ALERT_3_G Missing FCF Reflection(s) Below Theta(Min)[Deg]=       4.18 Note
                0  2  0,
PLAT912_ALERT_4_G Missing # of FCF Reflections Above STh/L=  0.600        297 Note
PLAT913_ALERT_3_G Missing # of Very Strong Reflections in FCF ....          1 Note
               -2  1  2,
PLAT941_ALERT_3_G Average HKL Measurement Multiplicity ..........         2.6 Low
PLAT954_ALERT_1_G Reported (CIF) and Actual (FCF) Kmax Differ by .          1 Units
PLAT969_ALERT_5_G The 'Henn et al.' R-Factor-gap value ..........      3.501 Note
              Predicted wR2: Based on SigI**2  4.01 or SHELX Weight 13.12
PLAT978_ALERT_2_G Number C-C Bonds with Positive Residual Density.          0 Info
```

```
   0 ALERT level A = Most likely a serious problem - resolve or explain
   0 ALERT level B = A potentially serious problem, consider carefully
  10 ALERT level C = Check. Ensure it is not caused by an omission or oversight
  29 ALERT level G = General information/check it is not something unexpected

   2 ALERT type 1 CIF construction/syntax error, inconsistent or missing data
  15 ALERT type 2 Indicator that the structure model may be wrong or deficient
  11 ALERT type 3 Indicator that the structure quality may be low
   9 ALERT type 4 Improvement, methodology, query or suggestion
   2 ALERT type 5 Informative message, check
```

Validation response form

Please find below a validation response form (VRF) that can be filled in and pasted into your CIF.

```
# start Validation Reply Form
_vrf_PLAT213_lcz014
;
PROBLEM: Atom F2              has ADP max/min Ratio .....        3.4 prolat
RESPONSE: ...
;
_vrf_PLAT220_lcz014
;
PROBLEM: NonSolvent   Resd 1   F   Ueq(max)/Ueq(min) Range        3.3 Ratio
RESPONSE: ...
;
_vrf_PLAT234_lcz014
;
PROBLEM: Large Hirshfeld Difference F1A      --C01Q     .       0.16 Ang.
RESPONSE: ...
;
_vrf_PLAT242_lcz014
;
PROBLEM: Low    'MainMol' Ueq as Compared to Neighbors of       C014 Check
```

```
RESPONSE: ...
;
_vrf_PLAT250_lcz014
;
PROBLEM: Large U3/U1 Ratio for <U(i,j)> Tensor(Resd    1)        2.1 Note
RESPONSE: ...
;
_vrf_PLAT342_lcz014
;
PROBLEM: Low Bond Precision on  C-C Bonds ..............    0.00961 Ang.
RESPONSE: ...
;
_vrf_PLAT906_lcz014
;
PROBLEM: Large K Value in the Analysis of Variance ......      4.889 Check
RESPONSE: ...
;
_vrf_PLAT911_lcz014
;
PROBLEM: Missing FCF Refl Between Thmin & STh/L=    0.600         17 Report
RESPONSE: ...
;
_vrf_PLAT934_lcz014
;
PROBLEM: Number of (Iobs-Icalc)/Sigma(W) > 10 Outliers ..          1 Check
RESPONSE: ...
;
# end Validation Reply Form
```

It is advisable to attempt to resolve as many as possible of the alerts in all categories. Often the minor alerts point to easily fixed oversights, errors and omissions in your CIF or refinement strategy, so attention to these fine details can be worthwhile. It is up to the individual to critically assess their own results and, if necessary, seek expert advice.

PLATON version of 17/09/2025; check.def file version of 17/09/2025

---

duplicate check

No duplication found

Datablock lcz014 - ellipsoid plot

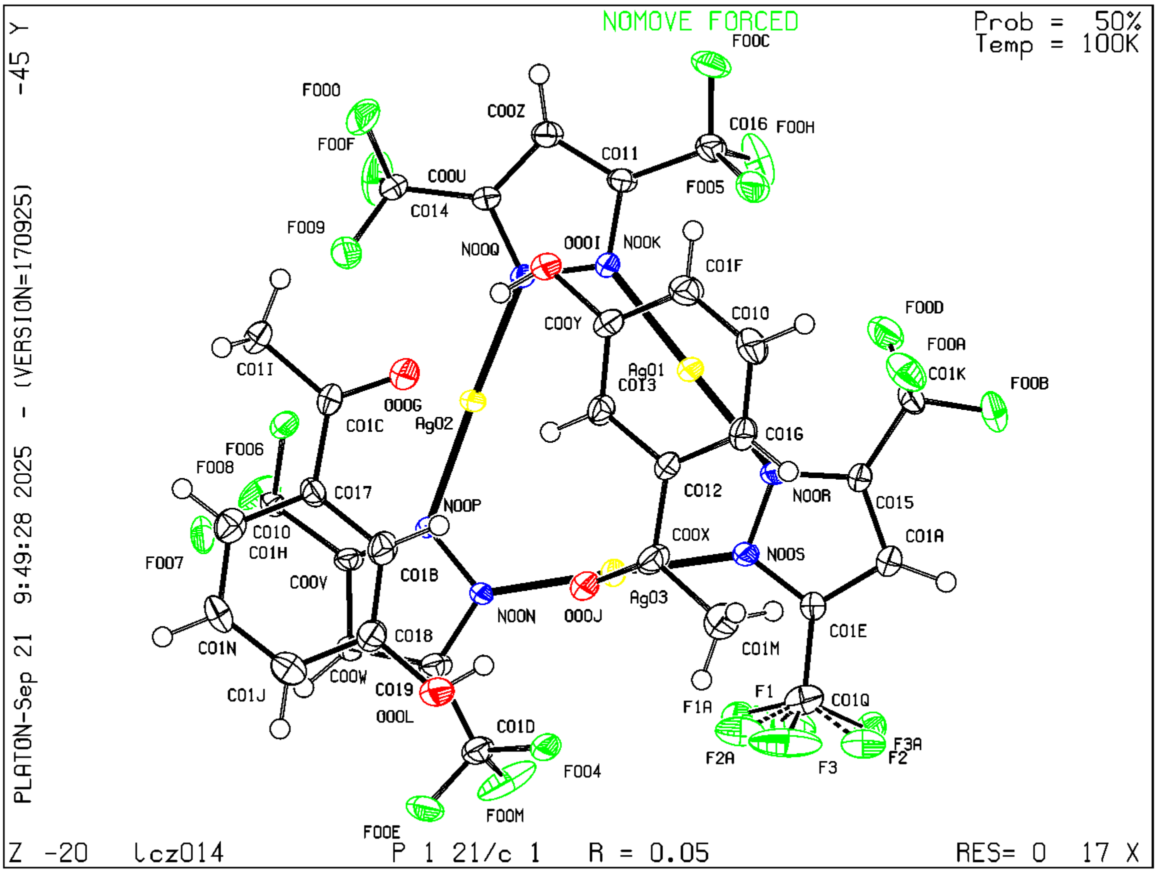

Supplement: Supplementary file 2 — Supporting File 2: anie72462‐sup‐0002‐DataFile.zip. [file ANIE-65-e1218503-s002.zip › supplementary_materials_crystallographic_data submit.zip!/cif_and_checkcif(1-114)/Ag3Pz3_14/Ag3Pz3_14_checkcif.pdf]
